# p90RSK pathway inhibition synergizes with cisplatin in TMEM16A overexpressing head and neck cancer

**DOI:** 10.1186/s12885-024-11892-9

**Published:** 2024-02-19

**Authors:** Abdulkader Yassin-Kassab, Suman Chatterjee, Nayel Khan, Nathaniel Wang, Vlad C. Sandulache, Eric H-B. Huang, Timothy F. Burns, Umamaheswar Duvvuri

**Affiliations:** 1 Department of Otolaryngology, University of Pittsburgh School of Medicine, Pittsburgh, PA USA; 2 UPMC Hillman Cancer Center, University of Pittsburgh Medical Center, Pittsburgh, PA USA; 3 Department of Otolaryngology Head and Neck Surgery, Baylor College of Medicine, Houston, TX USA; 4 Division of Hematology/Oncology, Department of Medicine, University of Pittsburgh, Pittsburgh, PA USA; 5 Veterans Affairs Pittsburgh Healthcare System, Pittsburgh, PA USA; 6 Smilow Research Center, 530 First Avenue, 801.b, New York, NY 10016 USA

**Keywords:** p90RSK, TMEM16A, Cisplatin, Resistance, BI-1870

## Abstract

Head and neck squamous cell carcinoma (HNSCC) constitutes one of the most common types of human cancers and often metastasizes to lymph nodes. Platinum-based chemotherapeutic drugs are commonly used for treatment of a wide range of cancers, including HNSCC. Its mode of action relies on its ability to impede DNA repair mechanisms, inducing apoptosis in cancer cells. However, due to acquired resistance and toxic side-effects, researchers have been focusing on developing novel combinational therapeutic strategies to overcome cisplatin resistance. In the current study, we identified p90RSK, an ERK1/2 downstream target, as a key mediator and a targetable signaling node against cisplatin resistance. Our results strongly support the role of p90RSK in cisplatin resistance and identify the combination of p90RSK inhibitor, BI-D1870, with cisplatin as a novel therapeutic strategy to overcome cisplatin resistance. In addition, we have identified TMEM16A expression as a potential upstream regulator of p90RSK through the ERK pathway and a biomarker of response to p90RSK targeted therapy in the context of cisplatin resistance.

**Supplementary Information:**

The online version contains supplementary material available at 10.1186/s12885-024-11892-9.

## Introduction

Head and neck squamous cell carcinoma (HNSCC) is the seventh most common cancer worldwide in 2022, accounting for 3% of all cancers and just over 1.5% of all cancer deaths in the United States [1]. Despite the recent advancement in our understanding of the disease leading to the development of novel therapeutic strategies, HNSCC related morbidity is still considered severe with a 5-year survival rate of 50% for the last three decades [2]. Current clinical strategies targeting HNSCC rely heavily on surgery, radiotherapy, chemotherapy, and molecularly targeted agents. Since its FDA approval in 1978, cisplatin has been widely used against a range of cancers, including HNSCC, and considered the standard first line treatment [3, 4]. Despite the significant efficacy of this treatment regime, high acute toxicity, increased treatment cost, and increased overall treatment time have been established as well-known drawbacks. In addition, acquired or intrinsic tumor resistance is often observed and combinations to overcome resistance are lacking. To overcome cisplatin-resistance, several combination therapeutic approaches have been developed by combining cisplatin with agents that can promote cisplatin efficacy against cancer cells [5–8], with limited success justifying the need for additional therapeutic options.

Here, we have characterized cisplatin acquired resistance in an array of HNSCC cell lines. We observed that hyperactivation of p90 ribosomal s6 kinase (p90RSK) signaling, a key downstream mediator of ERK1/2 signaling, as a critical component of acquired cisplatin resistance. Moreover, our results strongly suggest that the ERK/p90RSK signaling acts as central mediators of this resistance. Several members of the MAPK pathway, such as ERK, JNK, and p38 kinase, play critical roles in cell survival, proliferation, and migration of cancer cells [9]. RSK proteins are also involved in multiple cellular functions including cell survival, proliferation, cell cycle progression, and migration [10]. Although some previous studies have established p90RSK as a potent therapeutic target in affecting cell migration and proliferation of cancer cells [11, 12], the combinatorial activity of cisplatin with RSK inhibition is not widely explored, especially in HNSCC. Although TMEM16A overexpression is observed in around 30% of HNSCC and is associated with cisplatin resistance, apoptosis, poor morbidity [13], and can activate EGFR/ERK pathway [14, 15], the connection between TMEM16A and p90RSK has not been previously explored. Therefore, we chose to explore the connection between p90RSK and TMEM16A in the context of cisplatin resistance. In this study we identify p90RSK as a novel biomarker of cisplatin resistance and demonstrate that dual therapy of p90RSK inhibitor, BI-D1870, with cisplatin is synergistically lethal in high TMEM16A expressing HNSCC cell line models.

## Materials and methods

### Cell lines and reagents

HNSCC cell lines HN30 and HN31 were provided by Dr. Vlad Sandulache. HN5, Cal27, Cal33, UMSCC1, UMSCC9, and FaDu were purchased from American Type Culture Collection (ATCC). Te1, Te6, and Te9 were purchased from Novartis. OSC19 were purchased from Japanese Collection of Research Bioresources (JCRB). All cell lines were cultured in media as listed in Supplemental Table 1. The embryonic kidney cell line HEK 293T was purchased from ATCC and maintained in the laboratory following ATCC recommended growth medium. All cell lines were authenticated by autosomal STR validation profiling performed at IDEXX BioAnalytics, MO. Cisplatin resistant cell lines, HN30-R8 and HN31-P10, are described in [16]. HN30-R8 and HN31-P10 were cultured in growth media supplemented with 8 and 10 µM cisplatin, respectively. Cisplatin was purchased from EMD Millipore and dissolved in phosphate-buffered saline per manufacturer’s instructions. SCH772984 and BI-D1870 were purchased from Selleck Chemicals. Ganetespib was supplied by Synta Pharmaceutical Corp. (Lexington, MA). All primary antibodies were purchased from Cell Signaling.

### Cell proliferation assay

Three thousand cells per well were seeded in quadruplets for each treatment group in 96 well plates. After 24 h, cells were treated with indicated drugs and doses. Cell viability at 72 h was determined using the WST-1 Cell Proliferation Assay System (Takara Cat#MK400) following manufacturer’s protocol. IC50 values were calculated using GraphPad Prism software. Data generated was expressed normalized to untreated control. Combination Index for cisplatin and BI-D1870 was analyzed using Chou-Talalay method [17, 18] in the indicated cell lines. Each experiment was repeated two to three times independently.

### High throughput drug Screening

Drug screening was performed at the Gulf Coast Consortia using previously described methods [19], focusing on established and validated compounds with known activity in cancer cell lines. Briefly, using a 72-hour proliferation-based assay, coupled to high throughput quantification of cell number, we generated GR50 values for HN30 and HN30-R8 cells against a total of 88 compounds from the NCI AOD 5 compendium which are listed in Supplemental Table 2.

### Western blotting

After drug treatment, pellets were collected, and lysates were prepared as previously described [20]. At least 20 µg protein was loaded in each lane. Actin was used as loading control. Membranes were cut horizontally at appropriate molecular weights and developed independently to probe for various antibodies simultaneously. Uncropped images are available in supplementary files. Membranes were developed using chemiluminescence method on Gel Doc from Bio Rad. Densitometry analyses were performed using Image J software (NIH) available at https://imagej.nih.gov/ij/download.html. Each experiment was repeated two to three times independently.

### Retro- and lentiviral shRNA and cDNA overexpression

Four million HEK 293T cells were seeded in 25-cm^2^ flasks, and were transfected to generate lentiviral particles using a four-plasmid system as per the TRC Library Production and Performance protocols, RNAi Consortium, Broad Institute [21] and as previously described [22]. The pLenti CMV Puro DEST (W118-1) vector was obtained from Eric Campeau through Addgene (Addgene plasmid 17,452). The Ultimate™ ORFs (Invitrogen) for RSK1-4 and resulting constructs have been previously described [22]. All constructs were sequence verified. The ORF clone IDs of the constructs are– IOH46696 (RSK1.a, variant 1), IOH12130 (RSK1.b, variant 2), IOH63248 (RSK2), IOH3648 (RSK3), and IOH36120 (RSK4). Control or TMEM16A overexpressing cells were generated by viral transduction of UMSCC1 cells with viral pBABE-puromycin control or TMEM16A plasmid as previously described [15]. Cells were selected with puromycin-containing media for 48 to 72 h following transduction. HN30 cells were engineered to express control non-target shRNA (NT) or TMEM16A-targeting shRNA in a doxycycline-inducible manner as previously described [15, 23, 24]. Lentivirus expressing control, p90RSK1 and p90RSK2 were generated using plasmid [22] following protocol specified before. HN30-R8 were infected with the lentivirus diluted 1:4 with DMEM using 8 µg/ml polybrene for 24 h, followed by cisplatin treatment for 72 h. Viability was determined via WST-1 assay.

### In vivo studies

Female nude mice were injected subcutaneously with 3 × 10^6^ HN30 cells in 20% Matrigel (Corning) in bilateral flanks. Mice were randomized when the tumors became palpable into the following groups: vehicle, cisplatin, BI-D1870, and combination, with 5 mice per group. Cisplatin was dosed at 3 mg/kg, twice per week, and administered i.p. BI-D1870 was dosed at 50 mg/kg, i.p., 5 days/week. The volumes of tumors were measured every other day. All animals and data points were included in the analysis with a total *n* = 10 tumors per group. Animals were handled and euthanized according to University of Pittsburgh Institutional Animal Care and Use Committee (IACUC) protocol. Established humane endpoints included tumor length greater than 2 cm and tumor ulceration. Tumors were harvested, embedded in formalin, and given to the core facility for immunohistochemistry at the Pitt Biospecimen Core for pH2AX staining. Images were scanned at 40X magnification.

## Results

### Cisplatin EC50 values for HNSCC cell lines in vitro

**Fig. 1:**
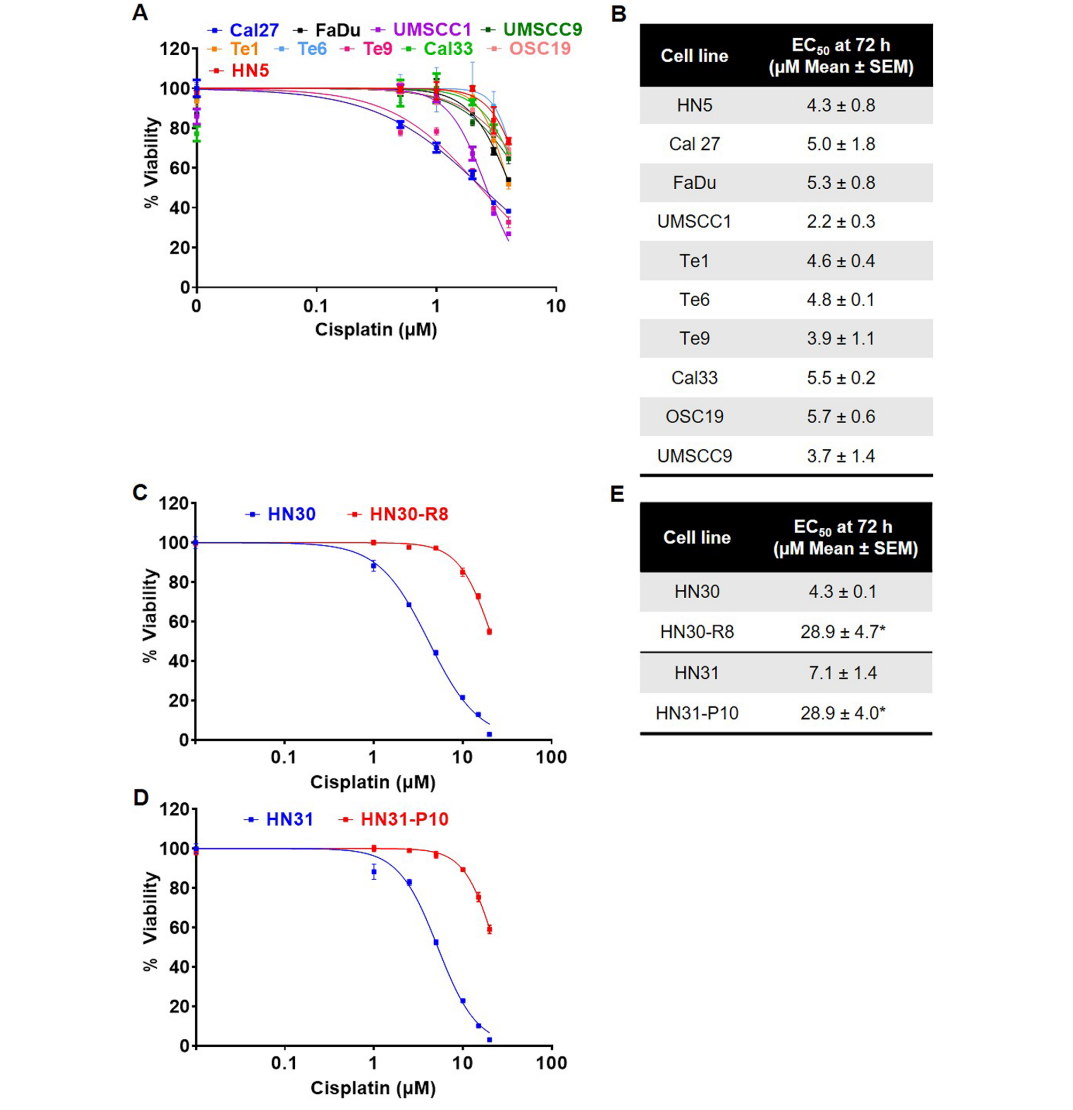
Cisplatin EC50 values for HNSCC cell lines in vitro. Cell proliferation assay at 72 h to determine EC50 values for cisplatin in the (**A**) indicated cell lines and (**C** and **D**) cisplatin resistant HN30-R8 and HN31-P10. Representative graphs from one experiment are shown. (**B** and **E**) Table indicating average EC50 value. Statistical significance was calculated using Student’s t-test. *p < 0.05

To begin with, we investigated the relative in vitro efficacy of cisplatin in a panel of human HNSCC cell lines. The representative cell viability curves after 72 h of cisplatin treatment are shown in Fig. 1A. Average EC50 values for each cell line are listed in Fig. 1B. We found UMSCC1 cells were the most sensitive with an EC50 value of 2.2 µM. Next, we concentrated on developing a workable model system to study the mechanism of cisplatin resistance in head and neck cancer. We used HN30 and HN31 cisplatin-resistant cells [16], HN30-R8, resistant at 8 µM cisplatin, and HN31-P10, resistant at 10 µM cisplatin. These cell lines were chosen because they have been shown to have inherently high TMEM16A expression and we were interested in exploring the role of this pathway in cisplatin resistance [25]. Figure 1C and D confirm significantly increased cisplatin resistance in vitro in the HN30-R8 and HN31-P10 cells compared to their parental counterparts. HN30 and HN31 cells had cisplatin EC50 values of 4.3 and 7.1 µM respectively, while the R8 and P10 cells both had an EC50 value of 28.9 µM (Fig. 1E). By utilizing this model of HNSCC, we could further investigate biological changes involved in acquired cisplatin resistance.

### Cisplatin resistant models display increased activation of p90RSK pathway

**Fig. 2:**
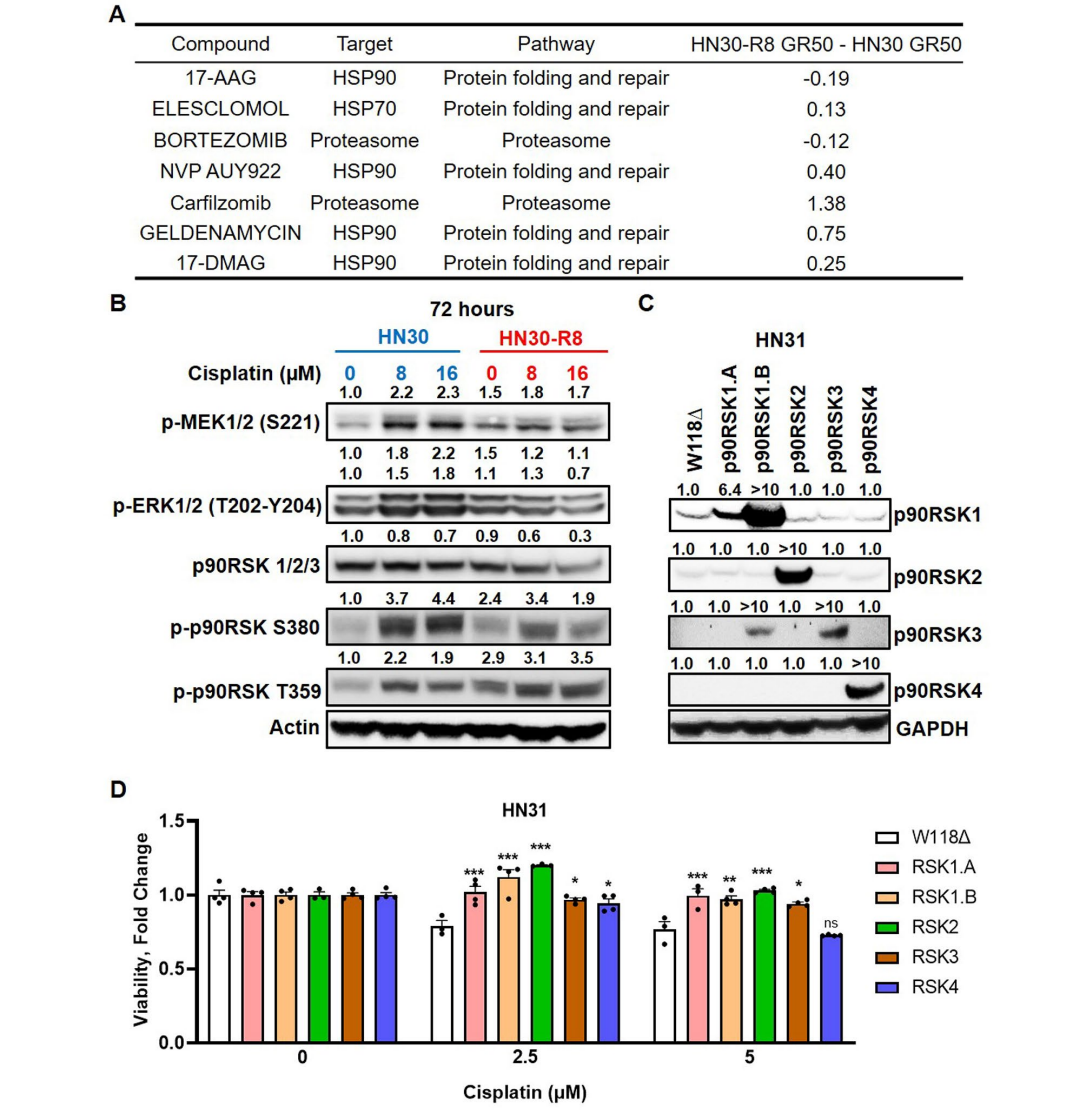
Cisplatin resistant models display increased activation of p90RSK pathway. (**A**) Table indicating top hits in drug sensitivities of HN30-R8 cells relative to HN30 cell. (**B**) Western blots of indicated cell lines after treatment with cisplatin for p-MEK, p-ERK, and p-p90RSK. One representative blot is shown. (**C**) Western blots of HN31 cells with induced overexpression of different p90RSK isoforms. One representative blot is shown. For B and C, fold changes of band intensities for respective protein are placed above the blot. (**D**) Viability of HN31 cells with overexpressing p90RSK isoforms treated with indicated concentrations of cisplatin. Comparisons are made between each p90RSK isoform to the wild type (W118∆) within each cisplatin treatment group. Statistical significance was calculated using two-way ANOVA with Dunnett’s multiple comparison test. *p < 0.05, **p 0.001, ***p < 0.0001

To identify potential therapeutic targets in the cisplatin resistant cell lines, a high throughput screen (HTS) was performed utilizing 88 drugs listed in the NCI AOD 5 drug set against the HN30 and HN30-R8 cells. The top hits are displayed in Fig. 2A with associated GR50 values indicating drug sensitivity. This drug screen indicated that cisplatin resistant cells may be sensitive to inhibition of the molecular chaperone HSP90. However, the resistant cells demonstrated cross resistance to the HSP90 inhibitor, ganetespib (Fig. S1A). Since we did not observe a significant response with ganetespib, (possibly because of off target effects of the drug), we decided to focus on HSP90 target, p90RSK, instead of a direct HSP90 inhibitor [22]. We had also previously shown that ganetespib resistance leads to dependance on the MEK/ERK/RSK pathway [22], and so we decided to explore this pathway in the HN30 and HN30-R8 cell lines. We first assessed basal expression of phosphorylated (activated) MEK, ERK, and its downstream target p90RSK in HN30 and HN30-R8 cells treated with 8 and 16 µM cisplatin for 72 h (Fig. 2B). We observed notable increase in the expression of p-MEK1/2 and p-ERK1/2 in cisplatin treated HN30 parental cells compared to R8. Additionally, we found that the cisplatin resistant R8 and P10 cells were more sensitive to ERK1/2 inhibition with SCH772984 than the HN30 and HN31 parental cells (Fig. S1B and S1C), although not statistically significant in the HN30-R8 cells, confirming the dependence of cisplatin resistant cells on ERK1/2. Activation of ERK1/2 leads to the activation of multiple downstream signaling molecules including the p90RSK (90 kDa ribosomal S6 kinase) family proteins. Taken together, Fig. 2A and B indicate that upregulation of the MEK/ERK/p90RSK pathway mediates cisplatin resistance in our model. To further confirm the role of p90RSK in contributing to cisplatin resistance, we expressed individual p90RSK isoforms in parental HN31 cells (Fig. 2C). Four human isoforms of p90RSK, 1–4, have been documented, among which isoforms 1 and 2 have been demonstrated to activate the mTORC1 complex activity [26]. Additionally, p90RSK proteins, especially isoform 2, has been shown to promote invasion and metastasis of human HNSCC cells [27]. We examined the HN31 expressing p90RSK isoforms for their response to 2.5 and 5 µM cisplatin (Fig. 2D). Evidently, expression of almost all isoforms induced significant cisplatin resistance at 72 h of treatment. Furthermore, using shRNA, we also knocked down p90RSK isoforms 1 and 2 in HN30-R8 cells (Fig. S2A) and observed the effect on cisplatin resistance. We found that knocking down these p90RSK isoforms resulted in a subtle, but significant, sensitivity to cisplatin in the resistant cell line (Fig. S2B), suggesting that p90RSK is the key-mediator in contributing to cisplatin resistance.

### Inhibition of the p90RSK pathway with BI-D1870 reverses cisplatin resistance in HN30-R8 cells in vitro

**Fig. 3:**
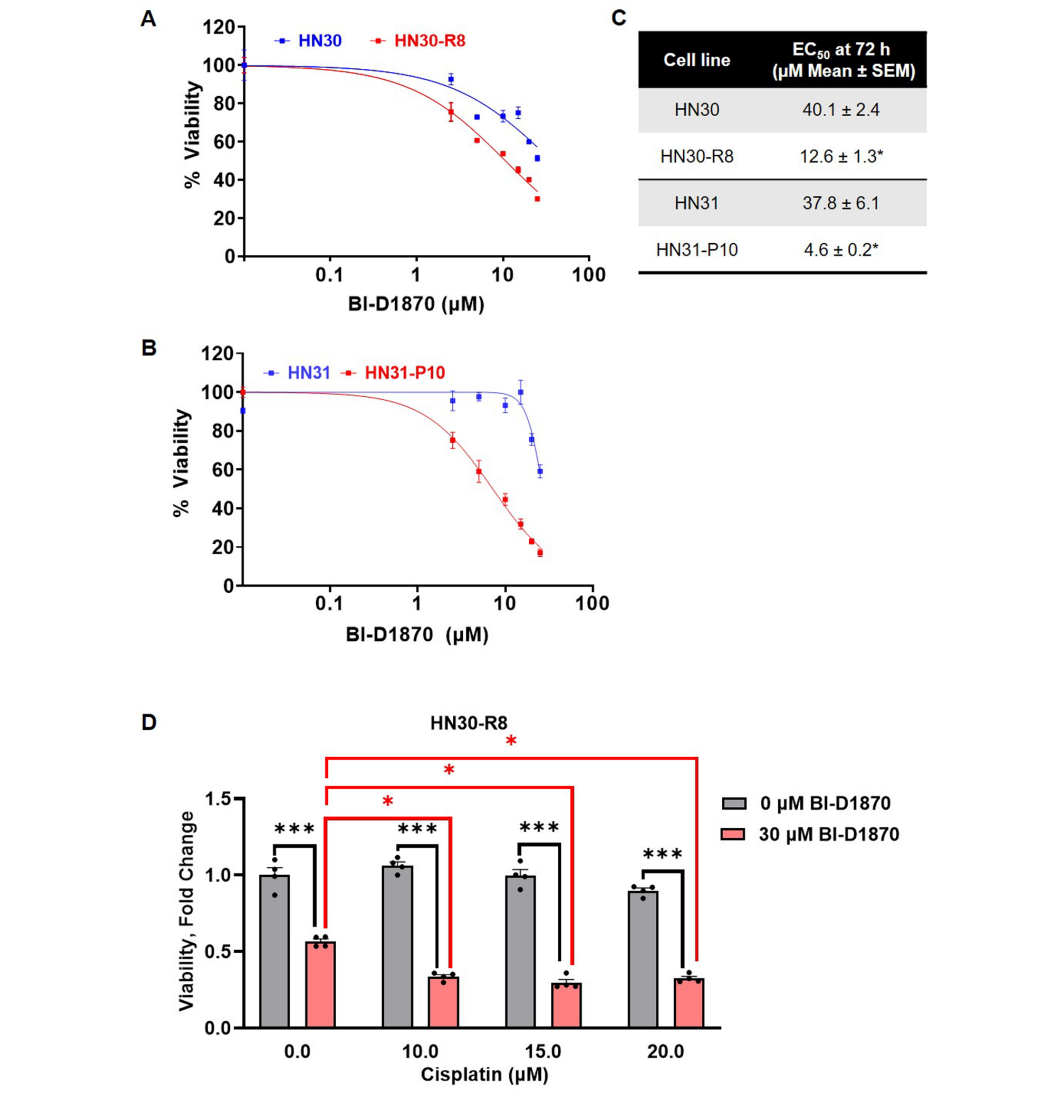
Inhibition of the p90RSK pathway with BI-D1870 reverses cisplatin resistance in HN30-R8 cells in vitro. (**A** and **B**) Cell proliferation assay at 72 h to determine EC50 values for BI-D1870 in the indicated cisplatin resistant cells and corresponding parental cell line. Representative graphs from one experiment are shown. (**C**) Table indicating average EC50 value for BI-1870. Statistical significance was calculated using Student’s t-test. *p < 0.05. (**D**) Viability of HN30-R8 cells treated with cisplatin and BI-D1870. Black asterisks compare the viability of cells with and without BI-D1870 within cisplatin treatments. Statistical significance was calculated using two-way ANOVA with Sidak’s multiple comparison test. ***p < 0.0001. Red asterisks compare the viability of cells treated with BI-D1870 alone to cells treated with cisplatin and BI-D1870. Statistical significance was calculated using two-way ANOVA with Tukey’s multiple comparison test. ***p < 0.05

Next, we sought to investigate whether p90RSK inhibition could serve as a therapeutic target in the context of cisplatin resistant model. Parental HN30 and HN31 cells and their cisplatin resistant derivatives, R8 and P10, were treated with 1–50 µM of an ATP-competitive inhibitor of RSK1/2/3/4, BI-D1870 [22, 28]. Viability curves are displayed in Fig. 3A and B. HN30 and HN31 cells demonstrated significantly less sensitivity to treatment, with R8 and P10 being over 3-fold more sensitive compared to respective parental cells (Fig. 3C). These data indicate that BI-D1870 is less active as single agent in the parental cells but is significantly cytotoxic in cisplatin resistant R8 and P10 cells, suggesting synthetic lethality of p90RSK inhibition in cisplatin resistant cells. To assess the combinatorial efficacy of cisplatin with BI-D1870, we treated HN30-R8 cells with a combination of the two drugs (Fig. 3D). Cells were treated with 10–20 µM cisplatin ± 30 µM BI-D1870 for 72 h. As expected, there was minimal cell death in the HN30-R8 cells treated with cisplatin alone. However, we observed significant cell death in the HN30-R8 cells at all concentrations of cisplatin in the presence of BI-D1870. Additionally, the combination of cisplatin and BI-D1870 induced more cell death than treatment with BI-D1870 alone. These results support p90RSK as a targetable signaling node, and the combination of cisplatin with p90RSK inhibitor, BI-D1870, as an efficacious combination to overcome acquired cisplatin resistance.

### TMEM16A expression is correlated with p90RSK in driving cisplatin resistance in human HNSCC

**Fig. 4:**
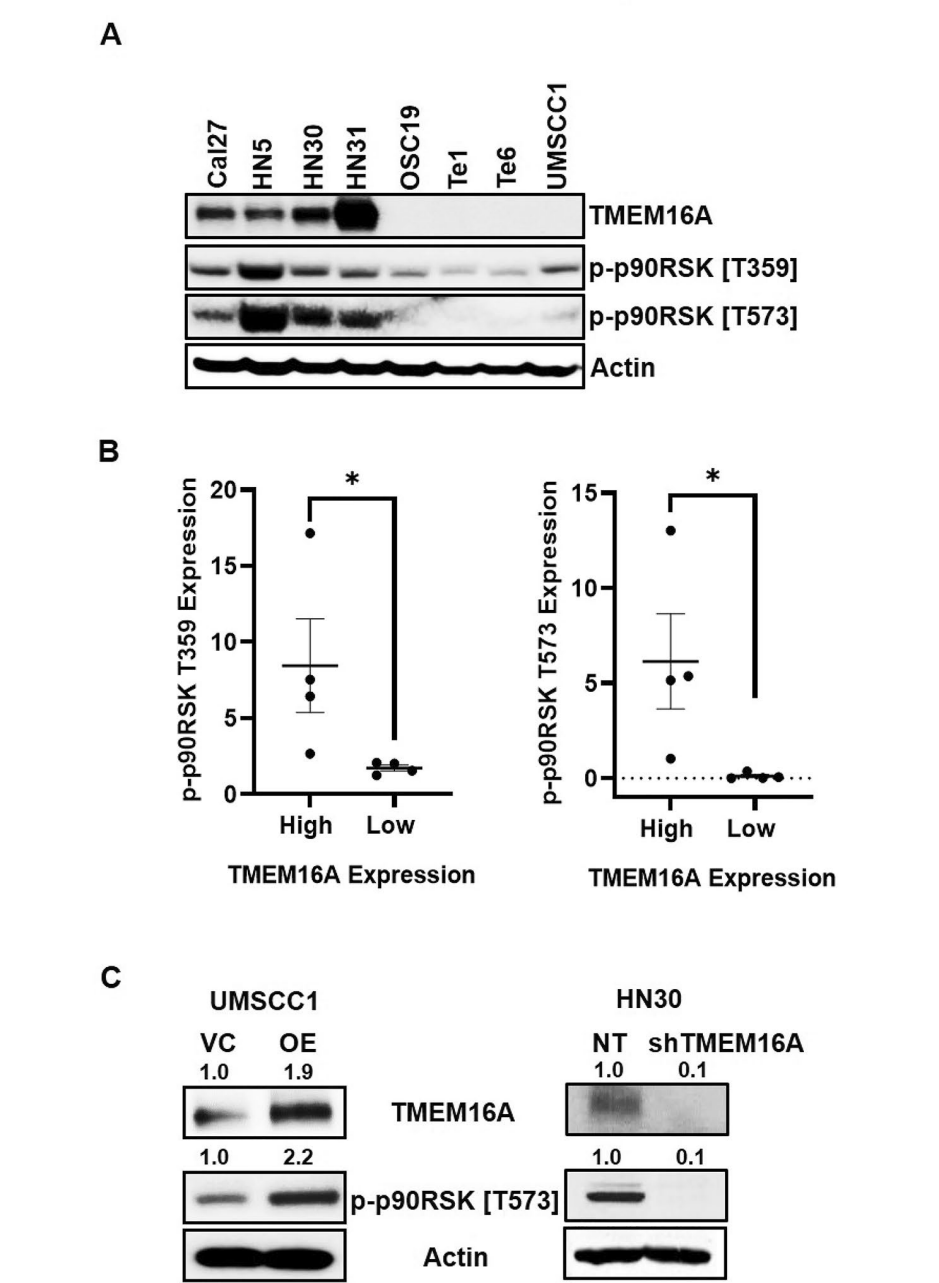
TMEM16A expression is correlated to p90RSK activation in cisplatin resistant HNSCC. (**A**) Representative western blot of indicated cell lines displaying basal expression of TMEM16A and phosphorylated p90RSK. (**B**) Dot plots quantifying protein expression of phosphorylated p90RSK and corresponding levels of TMEM16A expression. Dot plots from one representative experiment are shown. Statistical significance was calculated using Mann-Whitney test. *p < 0.05. (**C**) Western blots of indicated cell lines with modified TMEM16A expression and resultant phosphorylated p90RSK expression. Fold changes of band intensities for respective protein is placed above the blot. For A and C, representative blots from one experiment are shown

In HNSCC, TMEM16A overexpression has been associated with proliferation and tumor growth [15, 29], worse prognosis [15, 30] and undermining of clinical outcomes following platinum-based chemotherapy [31]. TMEM16A has been shown to induce MAPK signaling contributing directly to tumorigenesis and cancer progression [13, 15]. As p90RSK serves as an important downstream readout of ERK1/2 signaling; moreover, based on Fig. 2B, we sought to determine if TMEM16A has an impact on driving p90RSK mediated cisplatin resistance. To answer this question, we analyzed the expression of p90RSK in a panel of HNSCC cell lines with high TMEM16A expression (Cal27, HN5, HN30, and HN31) compared to a panel of cells with low TMEM16A (OSC19, Te1, Te6, and SCC1) (Fig. 4A). We observed a significant correlation between the TMEM16A expression level and active forms of p90RSK (Fig. 4B). To further clarify the correlation of TMEM16A with p90RSK, we used genetically manipulated cells lines for TMEM16A. Stable overexpression of TMEM16A in UMSCC1 cells led to increase in p90RSK, whereas knock down of TMEM16A in HN30 cells led to downregulation of active p90RSK (Fig. 4C). The effect on cisplatin resistance has been previously confirmed in TMEM16A overexpressing UMSCC1 cells that showed decreased apoptotic activity when treated with cisplatin [13], confirming the link between TMEM16A, cisplatin resistance, and downstream p90RSK signaling.

### TMEM16A expression can be used as a predictive marker for the efficacy of cisplatin and BI-D1870 combination in vitro

**Fig. 5:**
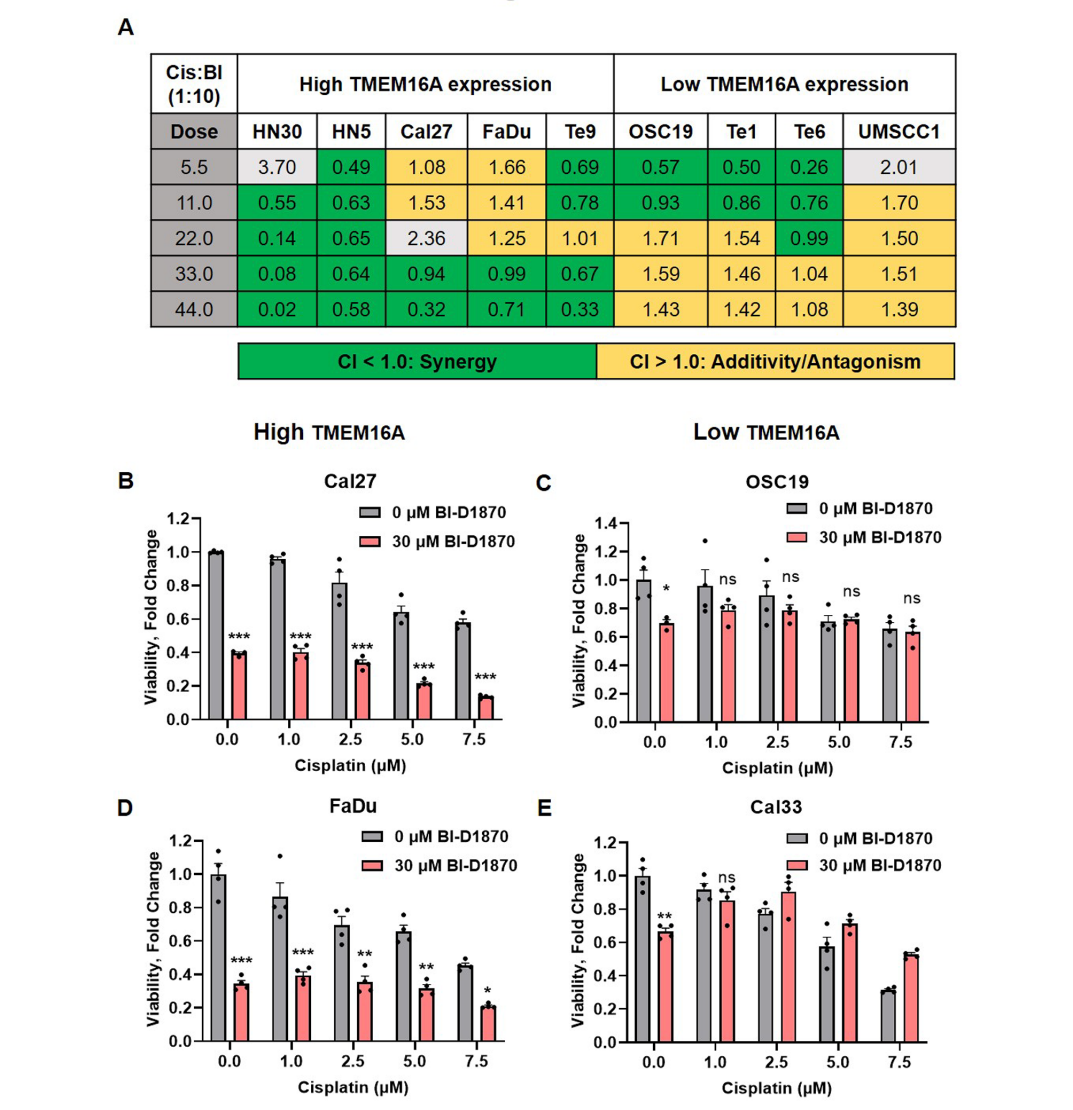
TMEM16A expression is a predictive marker for the efficacy of cisplatin and BI-D1870 combination in vitro. (**A**) Combination index (CI) values of indicated cell lines treated with increasing concentrations of cisplatin and BI-D1870 at a fixed ratio. CI less than 1.0 (green) indicates synergy, CI between 1.0 and 2.0 (yellow) indicates additivity, and CI greater than 2.0 (grey) indicates antagonism. This experiment was done twice. Data shown is the result of one experiment. (**B-E**) Viability of indicated cell lines treated with increasing concentrations of cisplatin with and without BI-D1870. Graphs from one representative experiment are shown. Comparisons are made between groups representing viability of cells with and without BI-D1870 within cisplatin treatments. Statistical significance was calculated using two-way ANOVA with Sidak’s multiple comparison test. *p < 0.05, **p 0.001, ***p < 0.0001

Next, we investigated p90RSK as a potential target to overcome cisplatin resistance. When BI-D1870 was used as a single agent, HNSCC cells with high TMEM16A expression were significantly more sensitive to treatment than those with low TMEM16A expression (Fig. S3A and S3B). In order to compare the efficacies of cisplatin and BI-D1870 combination in a high vs. low TMEM16A background, we performed synergy matrix analysis following Chou-Talalay method [18]. HNSCC cell lines in high vs. low TMEM16A categories were treated with cisplatin and BI-D1870 combination in 1:10 ratio for 72 h (Fig. 5A). Data represents CI (Combination Index) values for all cell lines treated with cisplatin and BI-D1870. In the low TMEM16A bearing cell lines, we observed an overall additive effect (CI value between 1.0 and 2.0) of the combination compared to strong synergistic efficacy (CI value less than 1.0) in high TMEM16A expressing cell lines. A Combination Index (CI) synergy curve in high TMEM16A expressing cells (HN30) displayed synergy at almost all Fraction Affected (Fa) values (Fig. S4A), while there was no synergy observed in the low TMEM16A expressing cells (UMSCC1) (Fig. S4B). To further establish TMEM16A as a predictive indicator of the efficacy of the combination of cisplatin and BI-D1870, we treated high TMEM16A expressing cells, Cal27 and FaDu, and low TMEM16A expressing cells, OSC19 and Cal33, with 0-7.5 µM cisplatin in the absence and presence of 30 µM BI-D1870 and measured cell viability after 72 h (Fig. 5B-E). As expected, we observed at least 50% cell death with the combination treatment in the high TMEM16A expressing cells (Fig. 5B and D) compared to cisplatin treatment alone. In the low TMEM16A expressing cells (Fig. 5C and E), the combination treatment did not induce differential cell death compared to cisplatin alone. Taken together, these data affirm TMEM16A as a potential biomarker for cisplatin and BI-1870 combination.

### Cisplatin and BI-D1870 combine to reduce tumor growth in TMEM16A expressing HNSCC in vivo

**Fig. 6:**
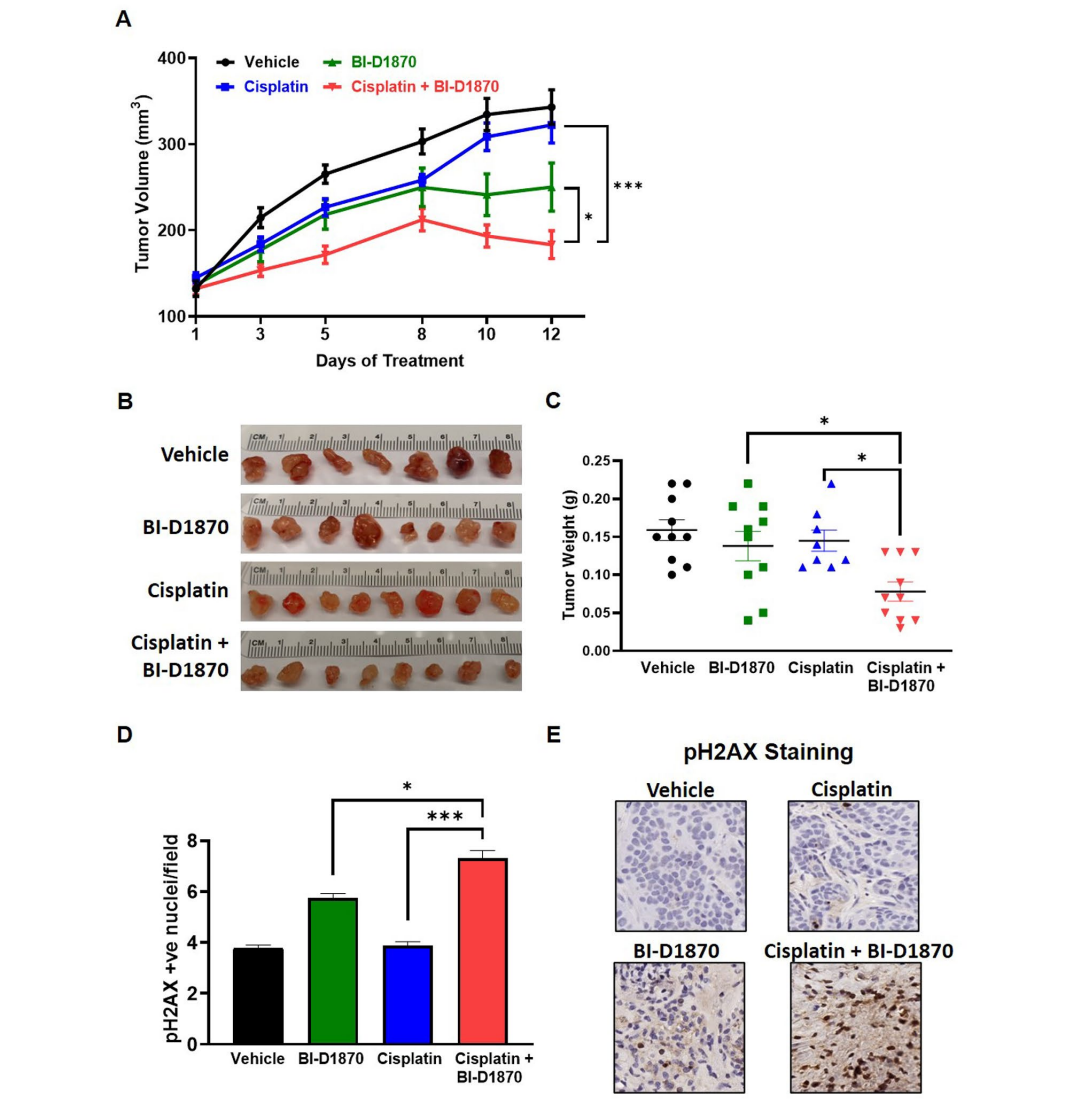
Cisplatin and BI-D1870 combine to reduce tumor growth in TMEM16A expressing HN30 cells in vivo. (**A**) Tumor volumes (mm^3^) in mice xenografted with HN30 cells after treatment with cisplatin and BI-D1870. Statistical significance for A was calculated using repeated measures two-way ANOVA with Tukey’s multiple comparison test. *p < 0.05, ***p < 0.0001. (**B**) Images of harvested tumors after treatment completion on Day 12. (**C**) Dot plot of the weights of harvested tumors (g) after treatment completion. Statistical significance was calculated using one-way ANOVA with Tukey’s multiple comparison test. *p < 0.05, ***p < 0.0001. (**D-E**) pH2AX staining and analysis in harvested tumors from each treatment group. Images were taken at 40X. Statistical significance was calculated using Fisher’s exact test. *p < 0.05, ***p < 0.0001

Finally, we tested our combination treatment in vivo using HN30 cells, which express high TMEM16A, in nude mice (Fig. 6A). At 12 days of treatment, tumor volume measurements showed decreased tumor growth in the mice treated with cisplatin and BI-D1870 combination group, when compared to mice treated with either drug alone. The experiment had to be culled after day 12 post treatment initiation as per our IACUC protocol since the tumors in the control group had ulcerated. Tumor images (Fig. 6B) and weights (Fig. 6C) confirm the efficacy of the combination treatment. The regression in the tumor volume observed in the combination group can be attributed to significant DNA damage as assessed by pH2AX staining in the harvested tumors (Fig. 6D and E). These data confirm that TMEM16A expression can be used as a predictive biomarker for the efficacy of adding BI-D1870 to standard cisplatin treatment.

## Discussion

Common chemotherapy treatments for HNSCC are cisplatin alone or cisplatin with 5-fluorouracil and docetaxel [32]. However, within the first 2 years of treatment, over 50% of patients with locally advanced HNSCC develop recurrence [33], an indication of treatment resistance. For this reason, many novel therapeutic drugs have been combined with cisplatin in studies attempting to increase cancer response rates [5–8]. Our goal was to elucidate targetable mediators of cisplatin resistance in HNSCC and to propose a novel combinatorial strategy to overcome resistance. We found that acquired cisplatin resistant models displayed upregulated MEK/ERK/p90RSK signaling. p90RSK is a key mediator in cell cycle regulation by phosphorylating and stabilizing components of the mitotic spindle that promote progression [34]. RSK isoforms also have anti-apoptotic effects, and their abnormal expression and activity is associated with multiple types of cancer [35, 36]. p90RSK inhibition monotherapy with BI-D1870 has been effective in HNSCC previously [37]. Additionally, the knockdown of RSK2 in ovarian cancer and the use of BI-D1870 in lung adenocarcinoma have proven to increase cisplatin sensitivity as well [38, 39].

We have previously shown that TMEM16A activates the Ras-Raf-MEK-ERK pathway and leads to increased phosphorylation of ERK1/2 in HNSCC [15]. It is therefore reasonable to hypothesize that TMEM16A also increases activation of p90RSK, a downstream target of ERK. Because of the role TMEM16A contributes to cisplatin resistance [13], it was of interest to investigate the association between TMEM16A and p90RSK as a potential tool to combat cisplatin resistance. Although the mechanism of TMEM16A to cisplatin resistance remains to be completely elucidated, we recently explored the role of lysosomes and TMEM16A [25]. In the current study, we confirmed that, not only is TMEM16A expression directly correlated to p90RSK activity, but that TMEM16A expression can be used as a predictive marker of the synergy between p90RSK inhibition and cisplatin.

BI-D1870 induces apoptosis by downregulating the MEK/ERK/p90RSK pathway, increasing cell cycle arrest, and generating ROS [37]. Cisplatin also exerts its cytotoxic effects through the induction of ROS and DNA adducts [40–43], enhancing cancer cell apoptosis [44]. Thus, it is not surprising that the combination of these drugs would enhance ROS generation, oxidative stress, and DNA damage, as evident from pH2AX analysis in the mice experiments. The translational significance of this study incriminates p90RSK in TMEM16A overexpressing tumors to surmount platinum resistance. Further work is required to establish TMEM16A specificity towards the cisplatin and BI-1870 combination. Additional future directions of this study include investigating the role of radiation or immune therapy in combination with BI-D1870 and cisplatin to improve oncologic outcomes.

### Electronic supplementary material

Below is the link to the electronic supplementary material.

Supplementary Material 1

Supplementary Material 2

Supplementary Material 3

## Data Availability

The authors confirm that the data supporting the findings of this study are available within the article and its supplementary materials.
