# Supplementary material for: p90RSK pathway inhibition synergizes with cisplatin in TMEM16A overexpressing head and neck cancer
Source: BMC Cancer. 2024 Feb 19;24:233. doi: 10.1186/s12885-024-11892-9 (PMC10875868; doi:10.1186/s12885-024-11892-9)
Supplement: Supplementary file 1 — Supplementary Material 1 [file 12885_2024_11892_MOESM1_ESM.pdf]

Supplemental Table 1

Cell line	Media	Source
HN30, HN31	DMEM +10%FBS +P/S + 1mM Sodium Pyruvate, 1x MEM Vitamin, 1x MEM NEAA	Vlad Sandulache [16]
HN5	DMEM/F12 + 10%FBS + P/S	ATCC
Cal27, Cal33, UMSCC1, UMSCC9	DMEM +10%FBS +P/S	ATCC
OSC19	DMEM +10%FBS +P/S	JCRB
FaDu	EMEM +10%FBS +P/S	ATCC
Te1, Te6, Te9	RPMI +10%FBS +P/S	Novartis

Supplemental Table 1. Growth media and supplements used for indicated cell lines.

Supplemental Figure 1

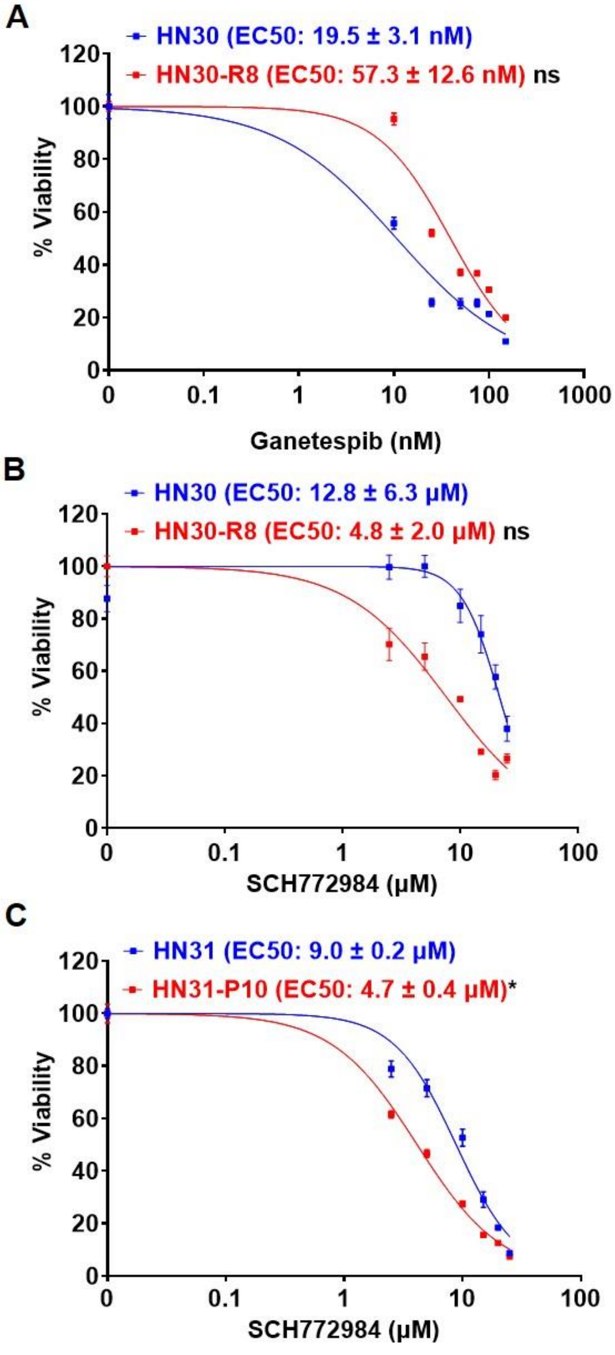

Supplemental Figure 1. Cell viability curves for HSP90 and ERK1/2 inhibitors. Cell proliferation assay and average EC50 values for (A) ganetespib and (B and C) SCH772984 in indicated cell lines. Representative graphs from one experiment are shown. Statistical significance was calculated using Student's t-test. *$p < 0.05$.

Supplemental Figure 2

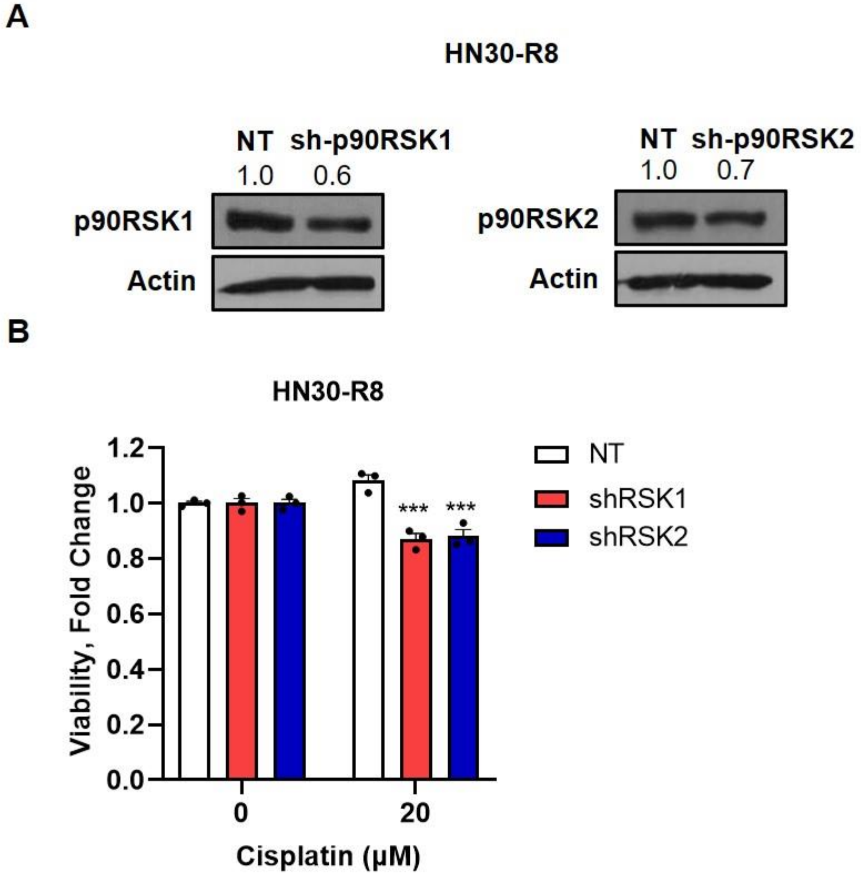

Supplemental Figure 2. Viability of HN30-R8 cells with shRNA knockdown of p90RSK isoforms 1 and 2.

(A) Western blots of HN30-R8 cells with shRNA knockdown of p90RSK1 and p90RSK2. Fold changes of band intensities for respective protein are placed above the blot. (B) Viability of HN30-R8 cells with p90RSK isoform knockdown treated with 20 μM of cisplatin. Comparisons are made between each p90RSK isoform to the non-target (NT). Statistical significance was calculated using two-way ANOVA with Tukey's multiple comparison test. ***p < 0.0001.

Supplemental Figure 3

A

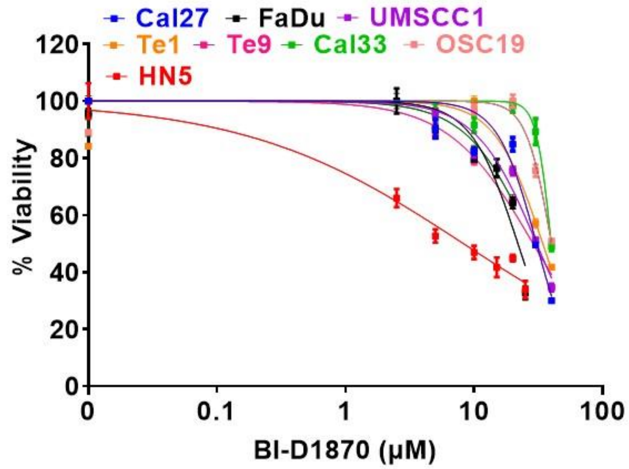

B

Cell line	EC_{50} at 72 h (μM Mean ± SEM)
High TMEM16A expression	
Cal27	22.1 ± 1.1
HN5	15 ± 4.7
FaDu	28.3 ± 4.5
Te9	18.2 ± 7.5
Low TMEM16A expression*	
OSC19	39.2 ± 1.8
Te1	35.0 ± 2.6
UMSCC1	30.3 ± 0.2
Cal33	39.8 ± 0.1

Supplemental Figure 3. Cell viability curves in HNSCC for BI-D1870.

(A) Cell proliferation assay for BI-D1870 in indicated cell lines. Representative graph from one experiment is shown. (B) Table indicating average EC50 value. Statistical significance was calculated using Student's t-test. *$p < 0.05$.

Supplemental Figure 4

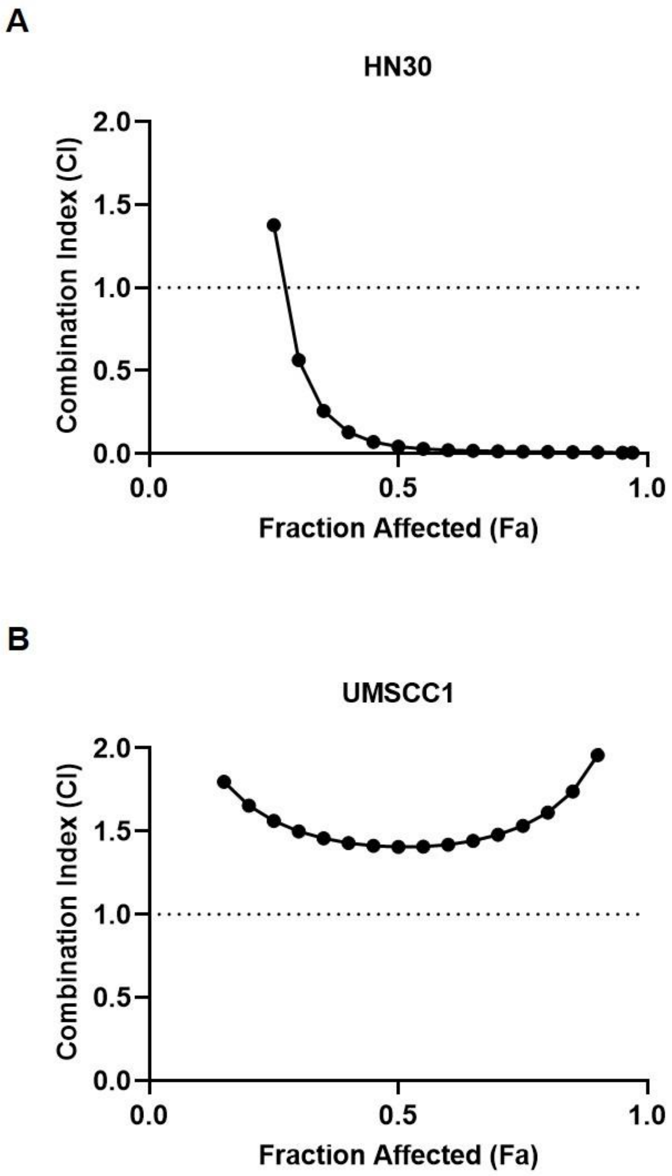

Supplemental Figure 4. Combination Index synergy curves for cisplatin and BI-D1870 in TMEM16A-high and TMEM16A-low cells.

Combination Index (CI) synergy curves for (A) HN30 (high TMEM16A expression) and (B) UMSCC1 (low TMEM16A expression) treated with cisplatin and BI-D1870. CI < 1.0 indicates synergy.
