# Supplementary material for: p90RSK pathway inhibition synergizes with cisplatin in TMEM16A overexpressing head and neck cancer
Source: BMC Cancer. 2024 Feb 19;24:233. doi: 10.1186/s12885-024-11892-9 (PMC10875868; doi:10.1186/s12885-024-11892-9)
Supplement: Supplementary file 3 — Supplementary Material 3 [file 12885_2024_11892_MOESM3_ESM.pdf]

Figure 2B Uncropped Western Blots

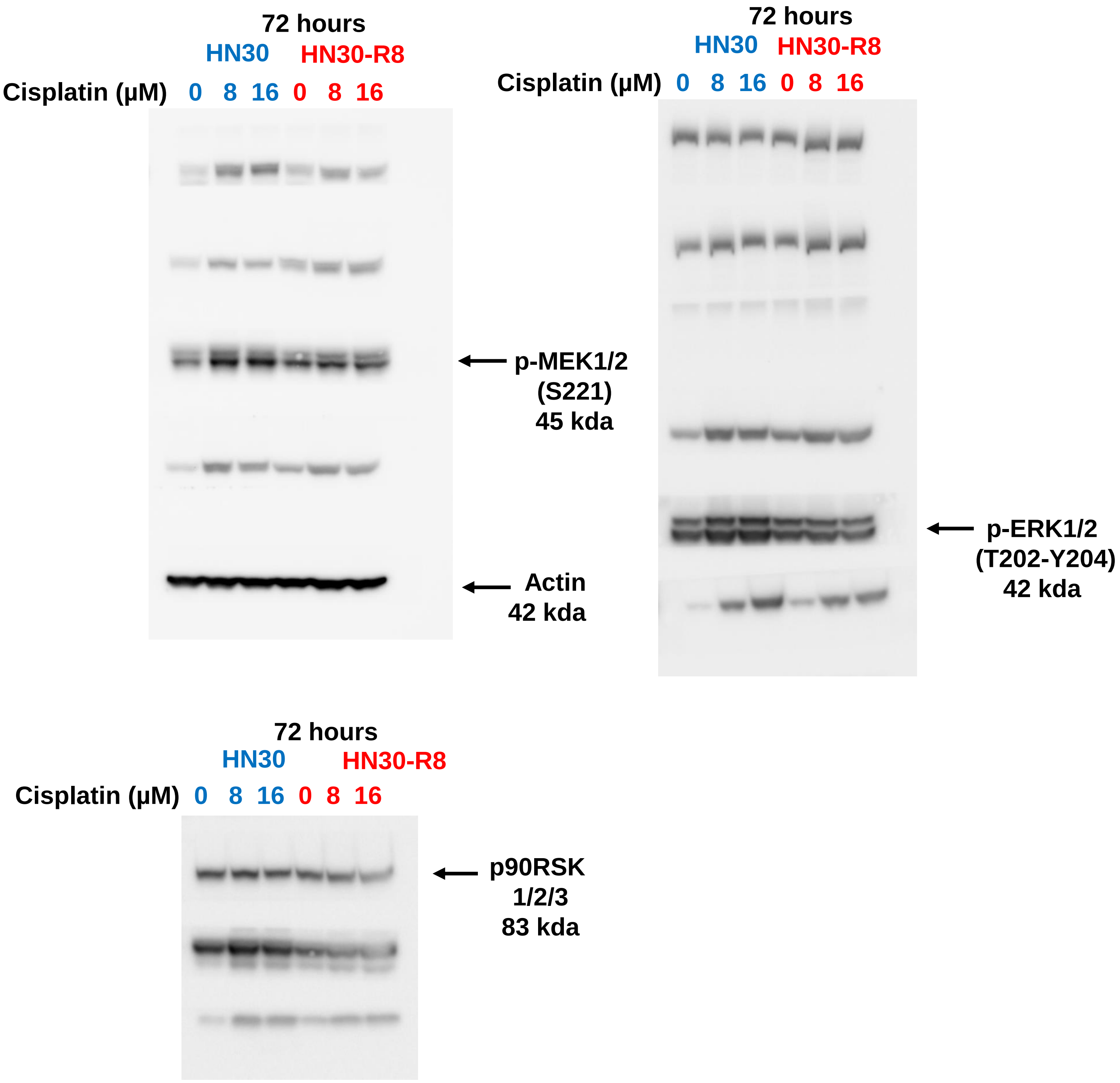

Figure 2B Uncropped Western Blots

72 hours
HN30 HN30-R8
Cisplatin (μM) 0 8 16 0 8 16

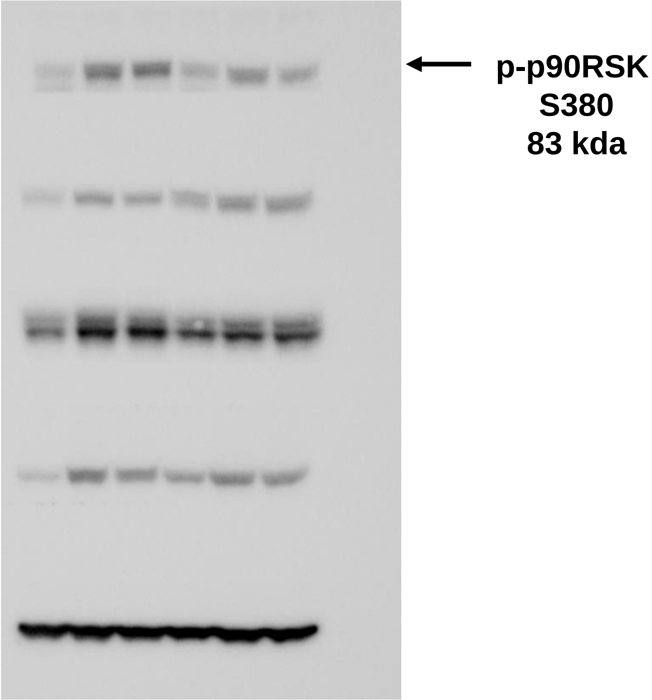

72 hours
HN30 HN30-R8
Cisplatin (μM) 0 8 16 0 8 16

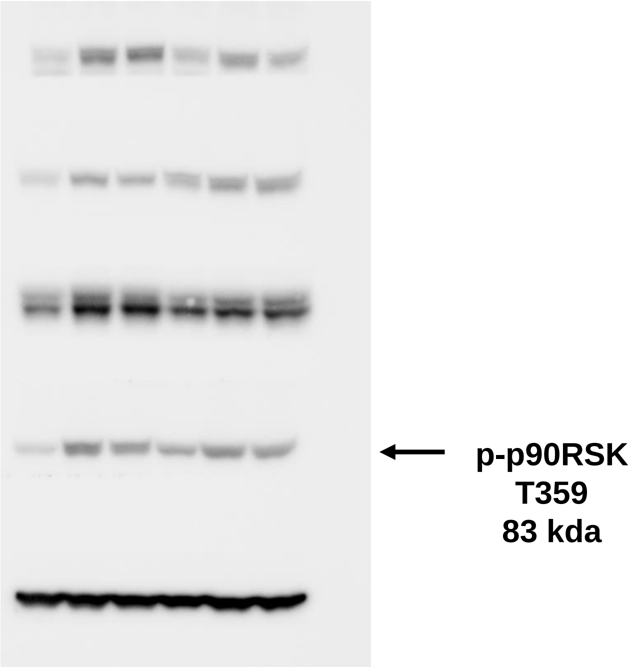

Figure 2C Uncropped Western Blots

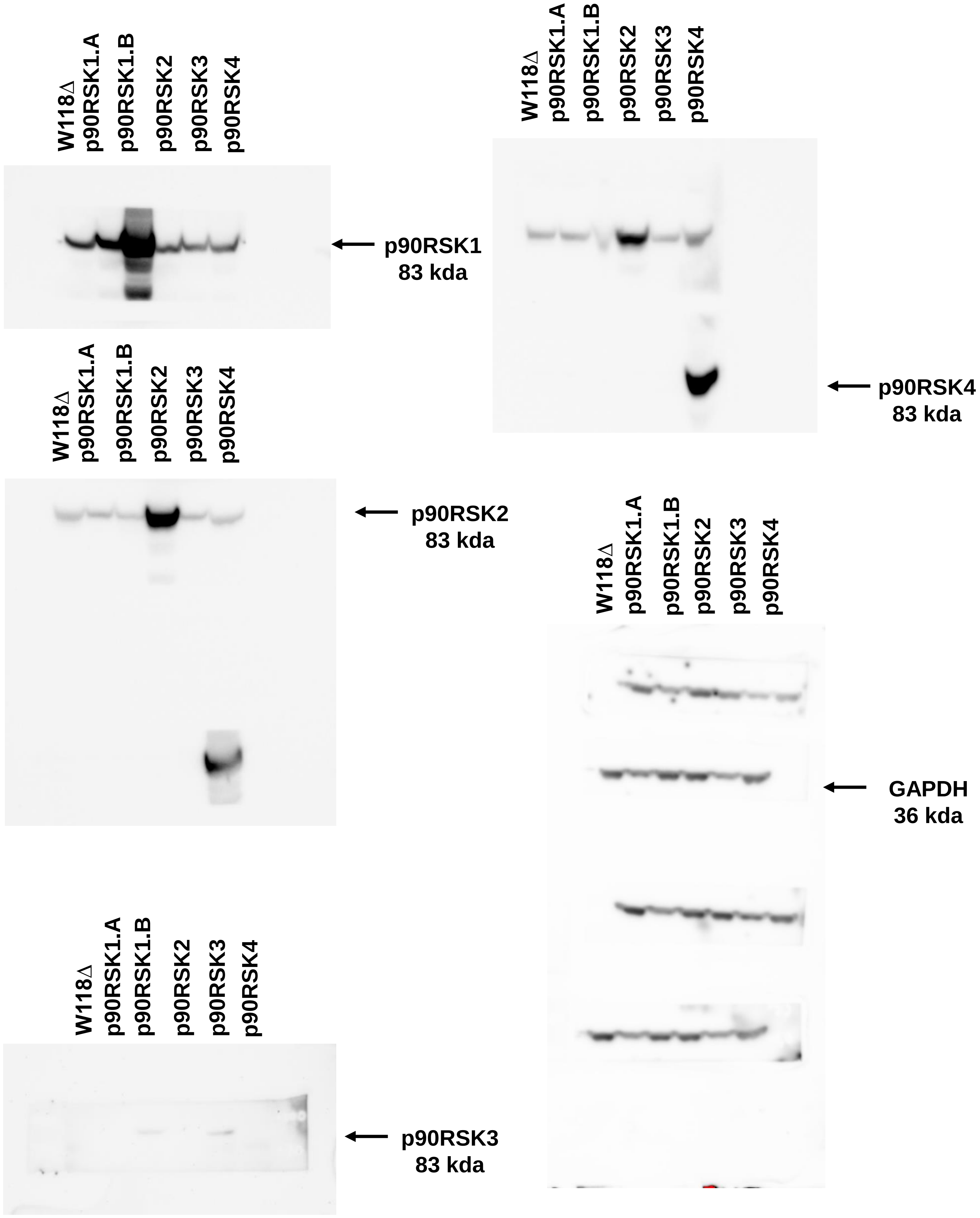

Figure 4A Uncropped Western Blots

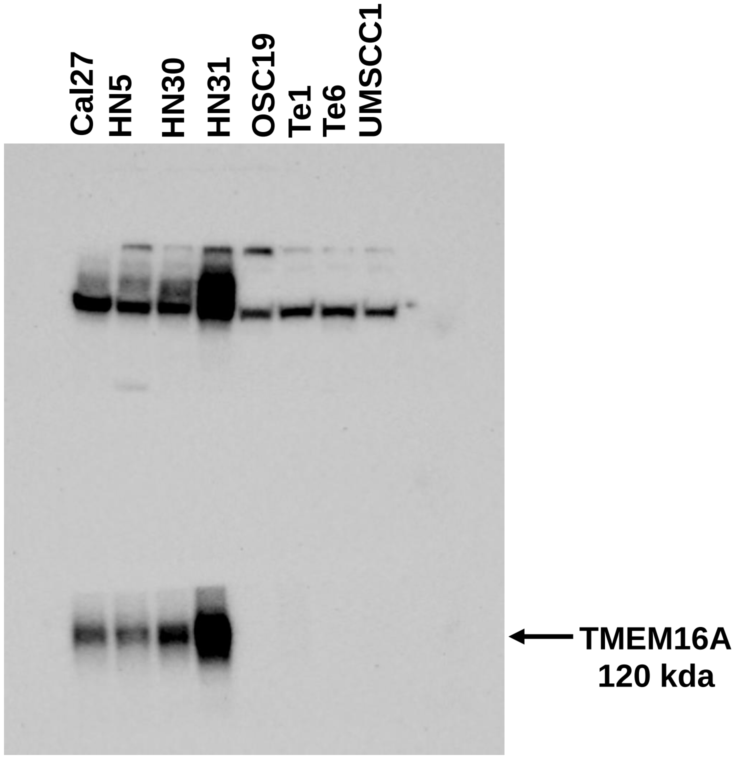

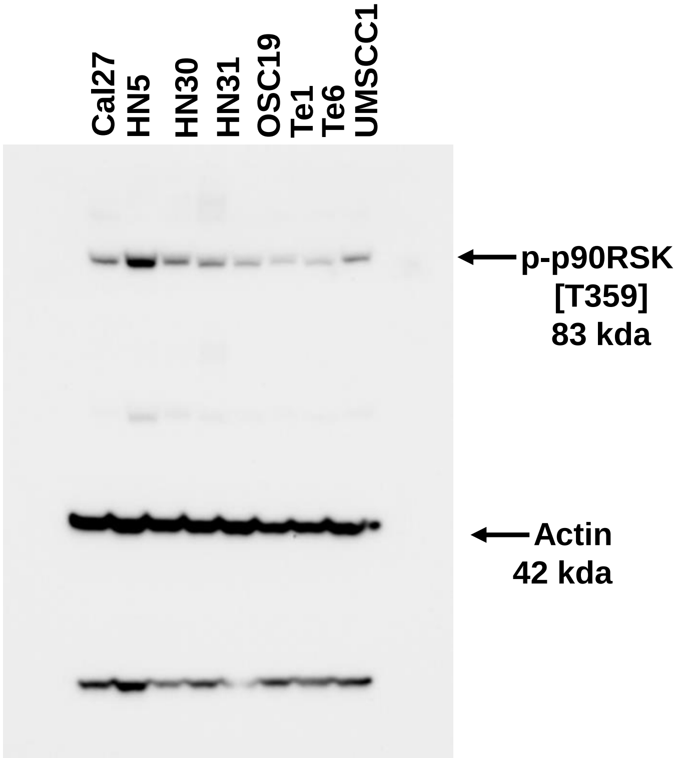

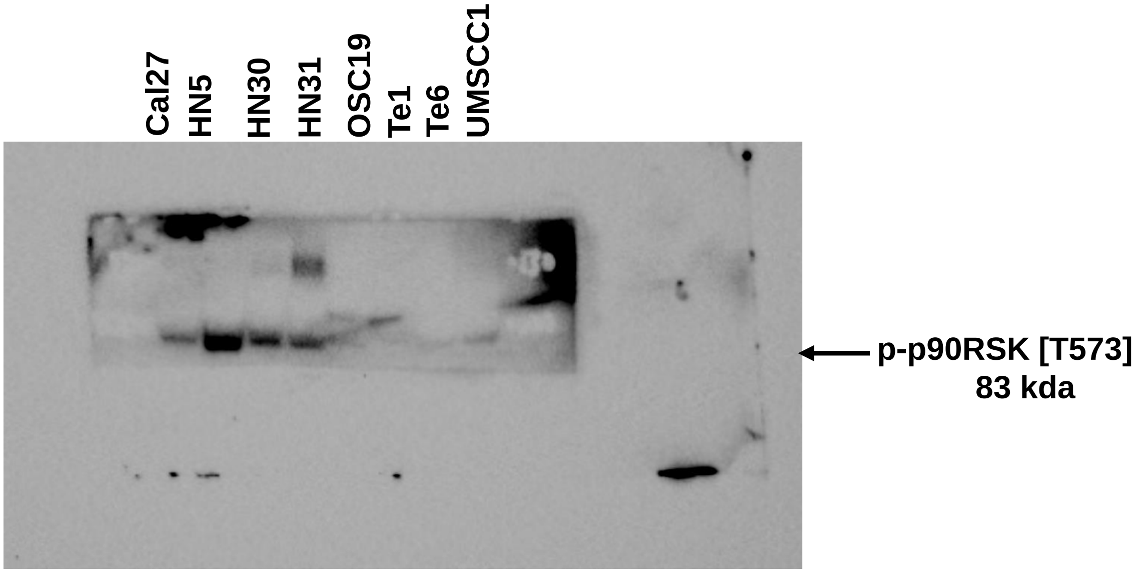

Figure 4C Uncropped Western Blots

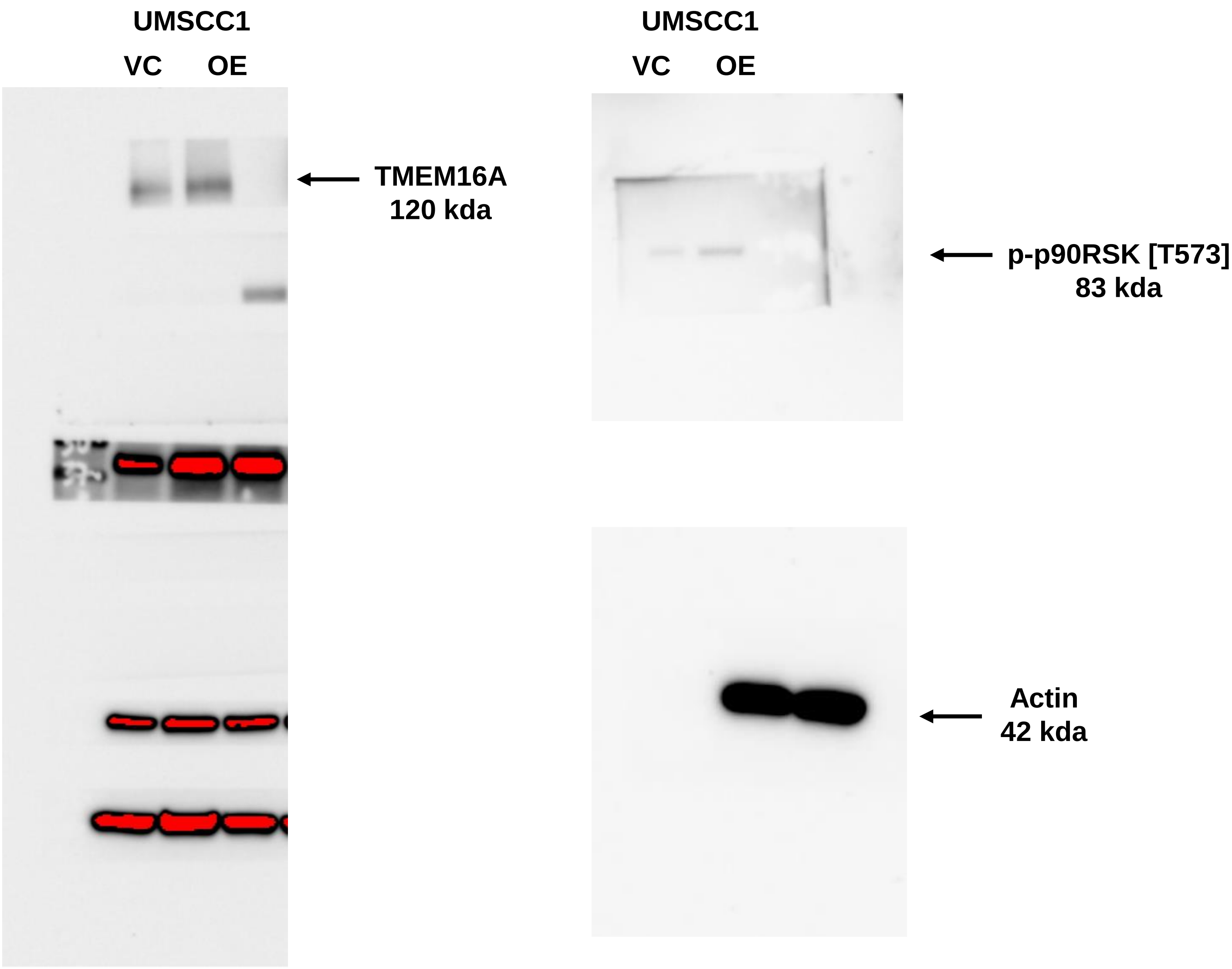

Figure 4C Uncropped Western Blots

HN30

shTMEM16A NT

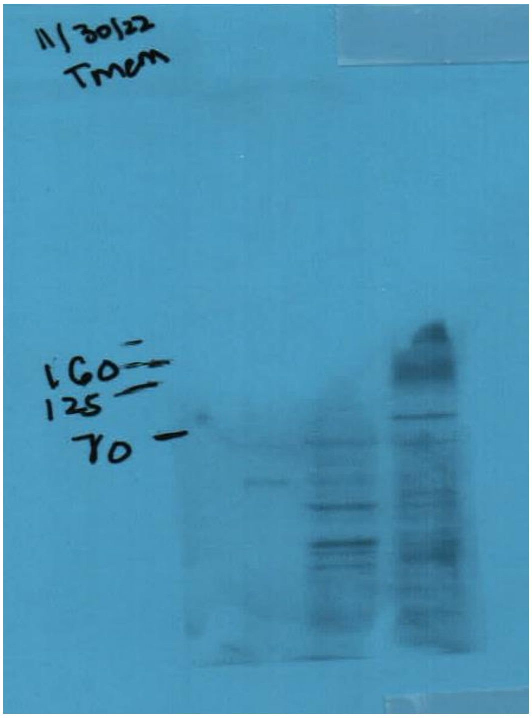

TMEM16A
120 kda

HN30

NT shTMEM16A

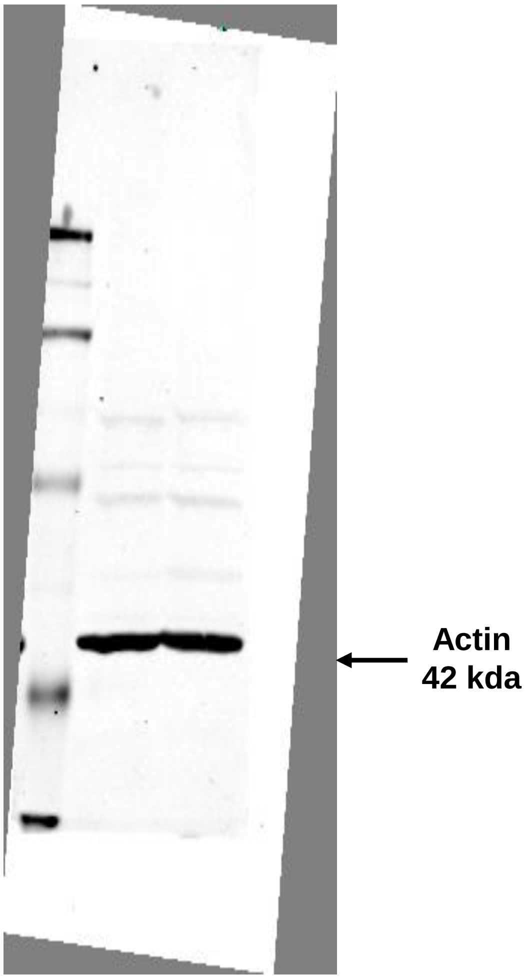

Actin
42 kda

HN30

NT shTMEM16A

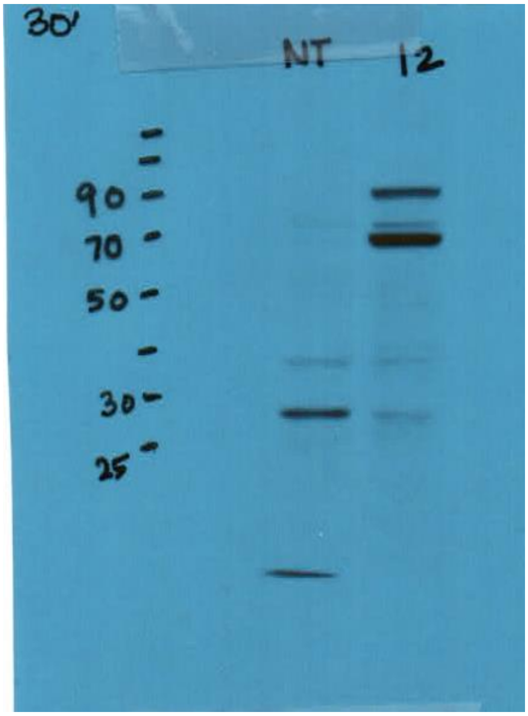

p-p90RSK [T573]
83 kda

Supplemental Figure 2A Uncropped Western Blots

HN30-R8

NT sh-p90RSK1

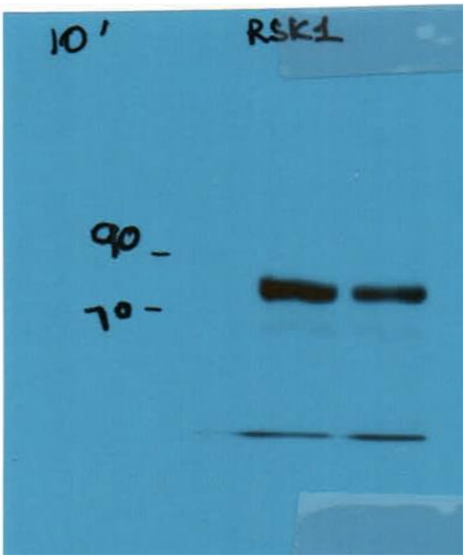

p90RSK1
83 kda

HN30-R8

NT sh-p90RSK2

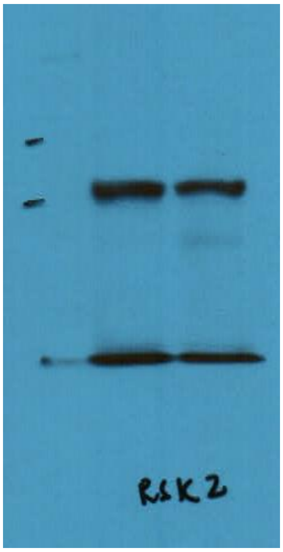

p90RSK2
83 kda

HN30-R8

NT sh-p90RSK1

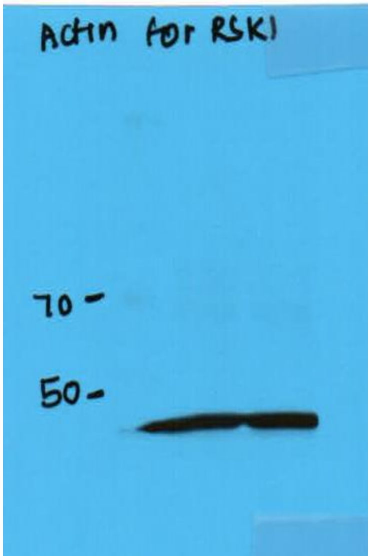

Actin
42 kda

HN30-R8

NT sh-p90RSK2

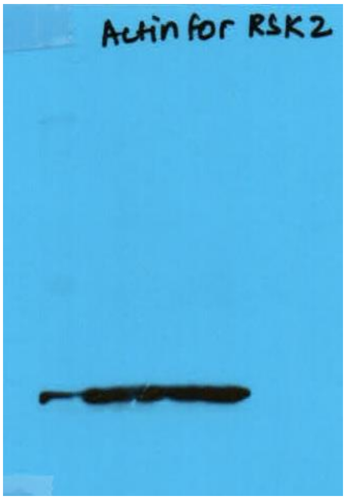

Actin
42 kda
